# Flipons, Noncoding Transcripts and Retroelements: A Universal Host Defense

**DOI:** 10.3390/ijms27198526

**Published:** 2026-09-24

**Authors:** Alan Herbert

**Affiliations:** Discovery, InsideOutBio, Charlestown, MA 02129, USA; alan.herbert@insideoutbio.com

**Keywords:** flipons, endogenous retroelements, noncoding RNA, dsRNA, Z-RNA, argonaute, reverse transcription, infection, immunity, interferon, cancer, lupus

## Abstract

Encoding genetic information involves both structure and sequence. Non-protein-coding (NCR) RNAs modulate host immune responses by guiding and assembling stress-specific protein effectors, with flipons enabling dynamic transitions between states. Small NCRs transcribed by both RNA Polymerases 2 and 3 normally modulate these pathways. The miRNAs, tRNA fragments, mitrons, piRNAs, and endo-small-interfering RNA products target the guide effector complexes to their targets. These NCRs filter the subset of RNAs that are translated, a process modulated by other NCRs that fine-tune RNA stability, localization, and kinetics through the modifications they direct. When these RNAs, or their targets, are dysregulated, host interferon responses are activated through the accumulation of uncapped triphosphorylated RNAs, dsRNAs, or flipon-encoded Z-RNAs. These responses are initially suppressed to avoid attacks on self-RNAs, but above a threshold, highly immunogenic pathways are unleashed. These same responses resist viral and other infections without pathogen-specific effectors or prior exposure. They also eliminate dysfunctional cells. These universal responses, when unresolved, contribute to a range of pathologies, increasing cancer, inflammatory diseases, and senescence. The review focuses on recent findings showing how these integrated host responses are driven by, and exploit, the retrotransposon invasion of animal genomes.

## 1. Introduction

Over the years, many different roles for noncoding RNAs in biology have been proposed, both good and bad, but no consensus exists. This contrasts with the coding genome, where the protein made from a transcript can be isolated, its properties determined, and its modifications mapped. Many regard this form of encoding, a triplet nucleotide cipher embedded in a DNA double helix capable of templating its own replication, as “the secret of life”. Yet many noncoding RNAs (NCRs) exist, and life could not exist without them. Examples include ribosomal RNAs, which catalyze the polymerization of amino acids into proteins, and small RNAs that regulate DNA readout, transcript processing, stability, and mRNA translatability. Other NCRs, such as 7SK, regulate RNA polymerase processivity, while 7SL promotes protein export across membranes. The classical view overlooks the essential role of NCRs in encoding genetic information and defending the genome from threats within.

## 2. Flipons

This different view of NCRs emerges from the idea that nucleic acids also encode genetic information by their structure. By adopting alternative conformations, NCRs can assemble distinct complexes with different functions. Such outcomes, for example, regulate immune responses that discriminate self from foe. Here, we explore this perspective. Sequences that encode alternative structures are called flipons [1]. They can transition from one conformation to another without a change in sequence or cleavage of the phosphate backbone. The alternative flipon DNA and RNA structures include left-handed Z-DNA and Z-RNA (referred here collectively as ZNAs), 3-stranded triplexes, and 4-stranded G-quadruplexes (GQ) (Figure 1A–D). Formation of each depends on particular sequence motifs, with ZNAs favored in particular by alternating pyrimidine–purine repeats, such as (dCdG:dCdG)_n_, triplexes by polypurine sequences, and G-quadruplexes by guanosine motifs capable of forming tetrads that stack on each other to form a quadruplex four-stranded helix [1]. ZNA and GQ motifs are enriched in promoters and endogenous retroelements (EREs) that are the focus of this discussion [2,3,4,5,6,7,8,9,10,11,12,13]. Of all these flipons, the evidence for biological function is strongest for ZNAs. The structure-specific Zα domain has provided the tools necessary to show this [14,15,16]. Through the work of many, the dependence of many biological pathways on ZNAs is supported by Mendelian genetic, biophysical, biochemical, and cellular biology evidence, as described throughout the manuscript.

Many factors determine when the flip from one structure to another will occur. These include structure-specific proteins; epigenetic modifications of both the nucleic acid and the proteins involved; topological stress generated by processive polymerases or helicases, or the ejection of nucleosomes from underwound DNA; and the stretching or twisting of RNA and DNA duplexes by tangles formed by pairing of complementary single-stranded RNA (ssRNA) sequences. The RNAs involved often affect multiple outcomes and are challenging to classify. Whether NCRs are long or short, repeat or non-repeat ssRNA, double-stranded RNA (dsRNA), or structured one way or another, they make no sense when evaluated by sequence alone (pun intended). However, flipons, as dynamic structural elements of the genome, are important to the evolutionary transition from self-replicating tinkers to cells [17].

**Figure 1:**
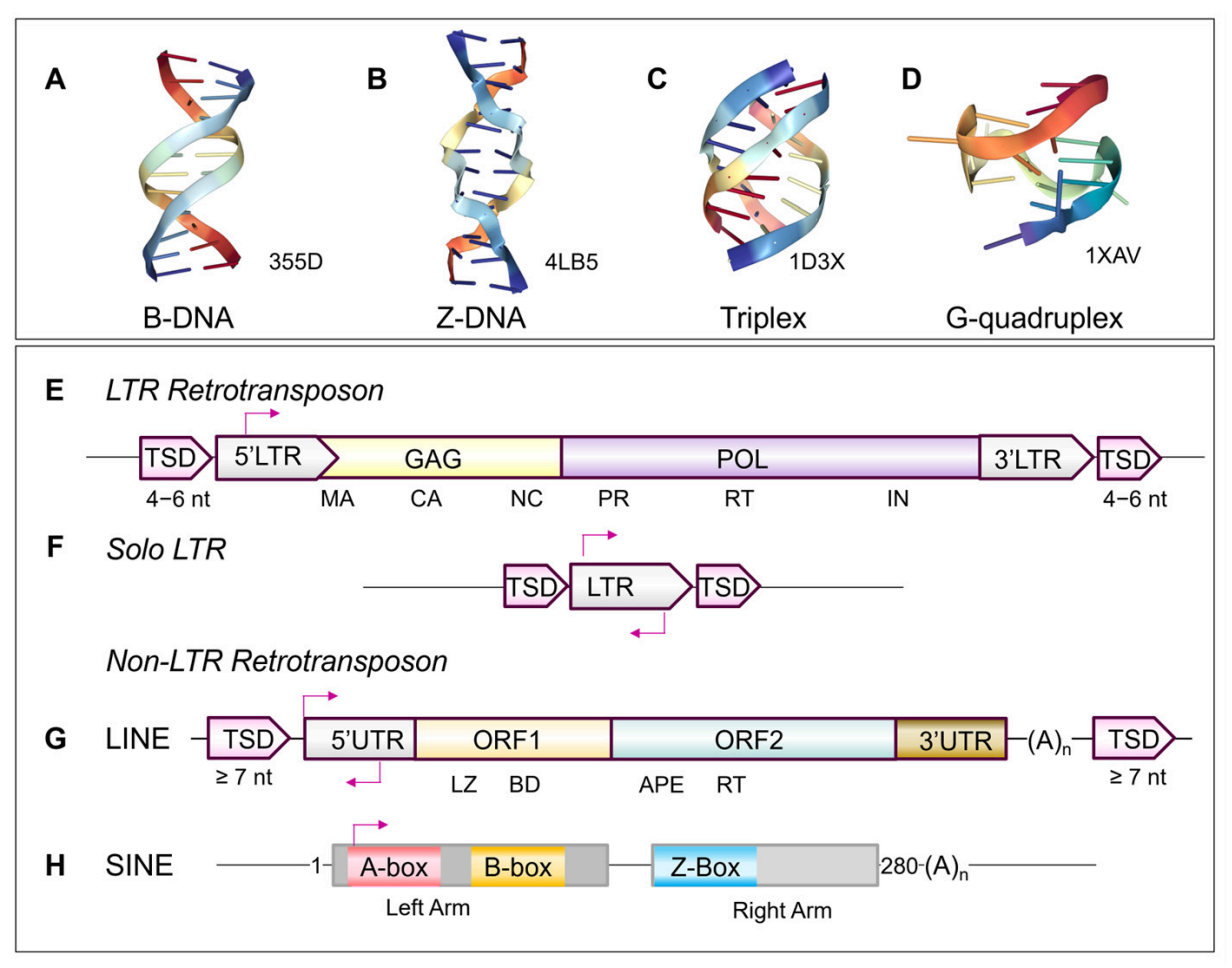
Sequence elements in the genome include flipons and retrotransposons. Flipons encode genetic information by adopting different DNA conformations as the context changes. The transition requires no sequence modification or backbone cleavage. Each structure helps seed different protein complexes. The alternative folds include: (**A**) Right-handed B-DNA. (**B**) Left-handed Z-DNA. (**C**) Three-stranded triplexes. (**D**) Four-stranded quadruplexes. The retrotransposons include: those with long terminal repeats (LTRs) (**E**), and solo LTRs derived from recombination of the 5′ and 3′ LTRs (**F**). Long terminal repeat (LTR) retrotransposons are composed of a group antigen (GAG) with matrix (MA), capsid (CA), and nucleic acid (NA) domains, a polymerase (POL) with protease (PR), reverse transcriptase (RT), and an integrase (IN) domain, but lack the envelope protein that is found in retroviruses. The terminal sequence duplication (TSD) is a duplication of the genomic sequence at the insertion site. Another class includes long interspersed nuclear elements (LINEs) (**G**) and short interspersed nuclear elements (SINEs) (**H**). A full-length LINE has two open reading frames (ORFs). ORF1 has a leucine zipper (LZ) and a C-terminal nucleic acid-binding domain (BD). The RNA polymerase 3 (RPOLIII) promoter binding sites (A- and B-box) are shown, along with the experimentally confirmed Z-box [7,18]. Only LTRs and LINEs encode the reverse transcriptase required to integrate their RNAs at new sites. SINEs compete for the LINE reverse transcriptases (modified from [19]).

To survive long enough, genomes require a sense of self, enabling them to defend against invasive replicants such as DNA and RNA transposons and viruses. These processes rely on NCRs, and their origins predate the emergence of sequence-specific nucleic acid-binding proteins and the embellishments they enabled, both in defense and in conquering new environs. Here, we describe how flipons encoded by NCRs enable real-time survival against threats and perturbations through a layered meshwork of protections.

## 3. Endogenous Retroelements and the Threat from Within

Here, the focus is not on viral and bacterial pathogens that attack from the outside. Instead, we will discuss the transposons that attack from within. Two classes of transposons exist: DNA and RNA (referred to here as EREs). They serve no purpose, but by spreading through a host genome, they disrupt active genes and pose an existential threat to a species. These threats have driven many adaptations that ensure host survival. The ideal solution is to eliminate transposons from a genome, but in some cases, these elements confer survival advantages. Even in bacteria with their lean genomes, transposons enable the horizontal transfer of advantageous traits from one individual to another. Here, the focus is on animal genomes where other solutions have left genomes filled with vast expanses of ERE-derived sequences.

DNA transposons and ERE did not always share the same evolutionary fate. For example, the human genome largely extinguished the DNA transposons about 37 million years ago [20]. These elements are maintained by a “cut-and-paste” mechanism. Although they comprise about 3% of the current sequence space, they are mostly incapable of transposition. Even so, the remnants of these elements are still targeted by Krüppel-associated box zinc finger proteins (KRAB-ZFPs) [21]. Over 74% of the sequences are classified as miniature inverted-repeat transposable elements (MITEs), which do not encode proteins. They retain the terminal inverted repeats that engage the transposases necessary for transposition. By doing so, they have in the past “cheated” active DNA transposons of the enzymes needed for their persistence, contributing to their mutual demise [20].

In contrast, ERE-derived sequences now occupy over 50% of the human genome. Their RNAs can capture the reverse transcriptases required for “copy and paste” retrotransposition immediately after the ribosome produces them. EREs use two distinct strategies to prime DNA synthesis. Long terminal repeat (LTR) retrotransposons use host tRNAs to prime the reverse transcriptase (RT). In contrast, the long interspersed nuclear elements (LINEs) cleave the host DNA and use the resulting ends as primers [22,23,24]. The latest estimate is that the human genome still contains 68 copies of active LINE L1 retrotransposases [25].

In both cases, other classes of ERE emerge that do not code for proteins. Solo LTRs arise by recombination between the 5′ and 3′ LTR ends, leading to loss of protein-coding sequences but retention of regulatory sequences. They can act as bidirectional promoters, driving transcription of adjacent regions. Like solo LTRs, short interspersed nuclear elements (SINEs) are noncoding (Figure 1E–H). They compete with LINEs for the RT as the protein emerges from the ribosome, ensuring that both persist. SINEs incorporate regulatory sequences from the host genome to ensure their own transcription, and disperse these throughout the genome [26,27]. Both solo LTRs and SINEs are enriched in transcription (TF) binding sites and in sequences, called flipons, that encode genetic information by the structures they form. As we will discuss, these ERE non-protein-coding sequences play key roles in host evolution.

Retrotransposon insertion into the genome is haphazard. In general, SINEs target active genes, while LINEs and LTRs accumulate in intergenic regions. These attacks trace back to the earliest eukaryotic progenitors. Immune responses were originally shaped to suppress LTRs, LINEs, and SINEs. These pathways now protect the host against a wide range of emerging threats. The invasive elements now defend the genome they previously ravaged. The focus is strictly on the role these elements play in host survival, a prerequisite for transmitting genetic information from one generation to the next. When these processes are dysregulated, they contribute to a range of pathologies, increasing cancer, inflammatory diseases, and senescence. Rather than classifying each retrotransposon or each noncoding RNA into a type and subtype that maps to a particular teleological function, I take a biological perspective. The text covers a wide range of topics and includes detailed supporting experimental evidence from the referenced publications. I begin with the NCRs produced by RPOL3, as their biology is quite impactful.

### 3.1. RPOL3 and Transcription

RPOL3 transcripts depend on A and B promoter sequences within a transcription Unit (TU), such as in SINEs (Figure 1H) [28]. Transcription relies on the RPOL3 TFIIIB and TFIIIC complexes. Importantly, the TFIIIC complex does not turn over. It acts as an anchor that ensures rapid RPOL3 turnover, enabling high-output states [29,30]. RPOL3 transcription is terminated by a poly(thymine) segment, usually 4–6 bases long, with the SENTAXIN helicase enforcing the stop. The size of the RPOL3 TU that lies between the transcription start site (TSS) and the TTS varies from ~70 to 90 nucleotides (nt) for tRNAs, ~100 to 300 nt for SINEs, ~300 nt for 7SL RNA, and ~100–200 nt for Vault RNA (vtRNA) and BC200 [31].

RPOL3 transcripts have an uncapped 5′ end with a triphosphate terminus. These ends are usually masked by the signal recognition particle SRP9/14 subunits [32]. This masking prevents binding of the retinoic acid-inducible gene I (RIG-I)-like receptor (RLR) gene products, which would otherwise activate an interferon response. The 3′ end is generally protected by that La protein that prevents digestion of the transcript by exonucleases [33].

### 3.2. Processing of RPOL3 Transcripts

The NCRs produced by RPOL3 are essential for normal cell function. They modulate the transcription and translation of many metazoan RNAs through a variety of NCR fragments [34,35,36,37]. Here, we first focus on tRNAs, then on SINEs, and their various roles in immunity.

tRNAs and their fragments: These NCRs include tRNA fragments that represent an ancient regulatory system dating back to the origin of eukaryotes (Figure 2A). A variety of tRNA fragments (tRFs) are involved in these pathways, including tRNA halves, quarters, and end pieces. These products have been extensively reviewed [36,38,39,40,41,42,43]. They arise in both Dicer-dependent and Dicer-independent ways through enzymes such as RNase-P and RNase-Z that act during pre-tRNA maturation. The tRFs have been assigned different functions depending on their single-stranded RNA sequence (reviewed in [41,43,44]). We focus on how the TRF duplex structure and length determine the effector pathways used, and how this impacts immune responses.

Small tRNA fragments that are duplex in character can be loaded into an RNA-induced silencing complex (RISC) by the Argonaute (AGO) maturation complex (AMC, Figure 2B) [40,46,47]. The AMC consists of the heat shock protein 90 beta (HSP90β), AGO, and the p23 cochaperone (encoded by the PTGES3 gene). All the components, including HSP90β, are abundantly expressed under normal conditions [48]. The geometry of tRNA fragments dictates their loading. Even after nicking, the tRFs remain duplex [49]. Structurally, they retain the tRNA L-shape, with the TψC stem stacked on the acceptor stem and the D-stem on the anticodon stem. Only the TψC-acceptor stem is of sufficient minimum length of 11–12 bases to fulfill the loading threshold of the AMC (Figure 2). Together with the single-stranded 3′-CCA tail overhang, the stem provides a template that resembles the 19- to 22-nucleotide microRNA duplex. Two nicks are required to free this stem from the tRNA and arm the AGO effector with an ssRNA guide. The tRFs preferentially associate with AGO1, 3, and 4 [50]. These argonautes are less stringent than AGO2 in their loading requirements and can accept guide RNAs with 5′ G and 5′ C ends, among other differences in their binding preferences [51]. The particular AGO loaded can have important biological consequences, affecting both the stability and translation of targeted RNAs through the complexes they form. For example, experimental studies reveal that tRF5-GluCTC induced by respiratory syncytial virus impairs post-transcriptional gene silencing in cells deficient in AGO1 or 4, whereas deletion of AGO2 and AGO3 has no effect [52].

The Dicer-generated fragments can also be loaded onto the RISC loading complex (RLC). This complex evolved later than the AMC and incorporates either the TAR RNA-binding protein (encoded by *TARBP2*) or the PKR-associated protein X (PACT, encoded by *PRKRA*) (Figure 2C,D) [53,54,55]. It was predominantly characterized by its association with microRNAs (miRNAs) [56]. In vitro, it is sufficient to load miRNAs onto AGO2 [57] but TARDP1 is not required in vivo [58]. The role of PACT is also challenging to analyze, as it suppresses PKR activation and the induction of inflammatory responses [59]. Recent evidence suggests that the RLC now cooperates with the AMC to increase the loading efficiency of various NCRs inside cells [47]. Another potential path to loading tRNA fragments onto Ago proteins would use pre-mRNAs as substrates for the classical pathway. However, the binding of La to the 3′ RNA end inhibits this outcome, with the exception of the pre-tRNA-Ile (Ile-TAT-2-3) in mice, which has low affinity for the LA protein. Processing of this pre-tRNA can then generate either the tRNA or mmu-miR-1983 [60].

In contrast, the 8- to 9-base-pair D-Anticodon stem is structurally too short for high-efficiency AMC loading [47]. Instead, modifications that shield them from exonuclease digestion protect the short TRFs they produce, allowing them to serve other roles. For example, TRFs that incorporate internal tRNA fragments can inhibit tRNA^Arg^ and tRNA^Ala^ deamination by Adenosine deaminase acting on transfer RNA (ADAT) [61]. The fragments produced can inhibit protein binding to their targets. An example is the 39-base-pair trf3e fragment produced from the 3′ end of tRNA^Glu^. The TψC stem–loop incorporates a 9-nucleotide sequence that binds nucleolin with high affinity (120 nM). The interaction prevents nucleolin-mediated inhibition of p53 protein translation [62]. In contrast, tRNA^val^ fragments directly bind the chaperone molecule eukaryotic translation elongation factor 1 alpha 1 (EEF1A1) to promote nuclear translocation. The subsequent activation of the mouse double minute 2 homolog (MDM2) ligase, which ubiquitinates p53, leads to its destruction [63]. Fragments can act in other ways by directly binding to the ribosome, as shown for the 5′ end of archaeal valine tRNA (Val-tRF) from *Haloferax volcanii* [64].

The tRNAs can contribute to cellular somatic defenses against ERE in somatic tissues. They inhibit the somatic spread of LTR retrotransposons, which depend upon tRNAs to prime reverse transcription. Interestingly, a 22 nt TRF3 can bind AGO and suppress translation of canonical transcripts. However, an 18 bp sequence that lacks the 3′ CCA tail cannot, as it is too short to engage AGO2 [47]. Notably, the 18 bp fragment that binds directly to primer sites inhibits LTR retrotransposition [38].

## 4. Cellular Stress and tRNA Fragment Structures

During stress, tRF production increases: angiogenin is released from inhibition by the Ribonuclease inhibitor RNH1, while RNase L is activated by the 2′,5′-oligoadenylate (2–5A) produced by interferon-induced oligoadenylate synthetases. The cleavage susceptibility and overall stability of NCRs depend heavily on the RNA modifications added during pre-tRNA processing. The outcome varies by tissue, reflecting the different tRNA isoforms and modification machinery expressed [65]. Experimentally, the long tRFs produced by angiogenin cleavage certainly increase during stress, but no new fragments are loaded onto AGO [66].

tRFs can form alternative structures, such as G-quadruplexes (GQ) and dumbbell dimers, which affect their persistence and function. For example, the GQ formed by tRNA^Ala^ and tRNA^Cys^ 5′ halves also inhibits translation and promotes stress granule formation [67,68]. Instead, the 5′ halves from tRNA^Gly^ and tRNA^Glu^ produced during stress have the potential to facilitate the translocation from the cytoplasm to the nucleus of factors that modulate alternative splicing and promote cell survival [69,70,71]. Notably, dimer formation masks the UUA sequence present in the Glu-5′tsRNA-CTC that drives mitochondrial import by leucyl-tRNA synthetase 2. In the mitochondria, the fragment inhibits the charging of mitochondrial leucine tRNA, disrupting mitochondrial translation and contributing to age-related neuronal degeneration [72]. Dimer formation also stabilizes the 5′ tiRNA-Gly-GCC. Dissociation of the dimer during stress protects neurons against excitotoxic necrosis through an antisense-mediated mechanism [73].

*Cellular Stress and AGO Loading*

During the heat shock response, AGO proteins localize to stress granules, where AGO2 is inactivated by ADP-ribosylation, thereby decreasing tRNA loading and RNA interference in human cells [74,75]. Also, despite increased production, HSP90β interactions with folded proteins reduce its availability, decreasing NCR loading through the AMC. These changes relieve the AGO-dependent suppression of the mRNAs encoding proteins that mediate stress responses in normal cells. A subset of tRFs may still load onto AGO during stress due to the increased tRNA levels, as occurs when tRNAs are overexpressed in normal cells [40]. 

Alternatively, the tRFs arising from enzymatic cleavage of tRNA may themselves directly reprogram translation and other cellular pathways [76]. A wide range of nucleases produce tRNA fragments, with angiogenin generating cyclic 3′ phosphates. This modification does not inhibit binding to AGO proteins but may instead be preferred for loading onto AGO2 [77]. In contrast, miRNA subsets are incorporated into the RISC during stress, including miR-210 (a hypoxia switch), miR-34a (DNA damage response), and mitrons, some of which form GQs that may modulate protein interactions [78,79,80,81,82,83].

## 5. RPOL3, RPOL2 and SINE Transcripts

SINE transcripts illustrate many other roles NCRs play in immunity. SINEs are transcribed by both RNA polymerase 3 (RPOL3) and RNA polymerase 2 (RPOL2), with the outcomes depending on the enzyme used. The RPOL3 readout echoes their origins. They are descended from highly expressed, RPOL3-transcribed 7SL RNA, which scaffolds the signal recognition particle (SRP) anchor essential for tethering ribosomes to the endoplasmic reticulum. SINE transcription by RPOL3 is strongly suppressed in differentiated cells, but increases dramatically under stress.

Most SINE transcripts in unstressed cells are instead synthesized by RPOL2, reflecting the preferential retrotransposition of SINEs into active genes over millions of years [84]. Often, insertion occurs in the reverse orientation relative to an existing family member, generating an inverted SINE repeat. During transcription, the repeats fold back on themselves to form dsRNA. Inverted repeats are most frequent in introns and the untranslated regions (UTRs) of mRNAs. These folds impact intron splicing, protein recoding, mRNA stability, and translation. They are enriched at the 3′ ends of genes, many lying just beyond transcription termination sites (TTS).

### 5.1. RPOL3 Regulates SINE Transcription Under Stress Conditions via Alu Elements

Surprisingly, RPOL3 mostly activates SINE transcription only under stress, reversing the suppression of EREs in normal cells that prevents their retrotransposition. This selective silencing of SINEs by facultative heterochromatin depends on the reversible histone marks written by the histone-lysine N-methyltransferase SUV39H1. Those SINEs that undergo DNA methylation are instead permanently consigned to constitutive heterochromatin. Their expression is not restored by 5-azacytidine inhibition of DNA methylation [85]. SINE repression in normal cells is highly efficient. Of the ~1.3 million Alu copies bound by the RPOL3 subunit TFIII3C, only ~1300 RPOL3 transcripts were detectable in an in vitro study of cell lines, with only 120 expressed in at least three cell types [86].

Epigenetic regulation renders SINE transcription by RPOL3 highly inducible. Over time, SINEs have acquired TF binding sites through mutation and deamination of methylated cytosines to thymine. One study reported that 34 TFs, out of the 1084 tested, bound to greater than 20% of Alu SINEs [26,27]. The SINEs also encode flipons that form ZNA, triplexes, and G-quadruplexes [7,18,87,88,89]. Cellular stresses, such as heat shock, also trigger expression from RPOL3-dependent Alu elements [90]. Similarly, activation is also initiated by adenovirus type 5 and herpes simplex type 1 infection [91,92]. The initial unpacking driven by heat shock factors (HSF) depends on a 13-base motif centered on position 221 of the Alu right arm that differs slightly from the consensus HSF1 and HSF2 GAA repeat sequences [93,94]. High levels of uncapped RNAs then trigger the RIG-I-directed interferon response to defend the cell.

### 5.2. RPOL3 Regulation of RPOL2 Transcription Under Stress Conditions

The activation of Alu transcription by RPOL3 by TFs, and during cell stress, also inhibits the RPOL2 transcription of many genes. This interaction is direct, with the Alu RNA blocking by disrupting contacts between RPOL2 and promoter DNA in preformed complexes [95,96]. Cryo-EM studies reveal that the Alu right arm structurally mimics the RPOL2 elongation complex. The interaction is of high affinity, with a K_d_ of ~20 nM [97]. During stress, Alus are rapidly expressed many hours before the transcription and translation of heat shock proteins, thereby protecting the cell in the interim from the activation of EREs that ectopically activate suppressed developmental pathways [90,98].

In other cases, RPOL3 instead promotes RPOL2 transcription. Overall, ~20% of active Alu elements are within 2 kb of an active divergently transcribed RPOL2, with 60% belonging to the Alu-S family [99,100,101]. Of these, ~2% to 3% are actively induced by RA, with the RA receptor binding to a direct repeat motif with a 2-base spacer (DR2) on the Alu left-arm. Around 100,000–200,000 SINEs have DR2 elements and are located close to RPOL2-transcribed genes, with 95% of RA-activated elements from the Alu-S subfamily of repeats. Over 18,000 of these Alu-DR2 SINEs are situated directly within a regulatory window of ±10 kilobases (kb) of the 5′ ends of more than 10,000 RPOL2-transcribed genes [102]. Activation of RPOL3 transcription likely turns these elements into enhancers that promote RPOL2 transcription of the associated gene [99,103,104]. The best-characterized genes responding to RA are those involved in stem cell maintenance, such as teratocarcinoma-derived growth factor 1 (TDGF1) and the homeobox protein NANOG [105]. RA also enhances expression of interferon-induced proteins involved in antiviral defenses [106].

After expression, DR2-induced Alu transcripts can be processed through a Dicer-dependent pathway, producing fragments of ~28–65 nt. After export to the cytoplasm, AGO3 binds the RNAs, which lack the strict length restrictions of other Argonaute family members [105]. Evidence suggests that AGO3-bound repeat-induced RNAs scaffold assembly of the Enhancer of Decapping Complex 4 (EDC4, also known as RCD8) to downregulate RPOL2 transcripts from the neighboring gene. The complex formed promotes mRNA degradation by Exoribonuclease 1 (encoded by XRN1), creating a feedback loop in which RPOL3 transcripts control RPOL2 mRNA levels. The AGO3-bound RPOL3 Alu transcripts engage complementary sequences in the 3′ UTR of TDGF1 and NANOG, terminating stem cell pluripotency. As reported in early studies of vitamin A, they also limit interferon responses [107].

## 6. RPOL3-Mediated Retrotransposon Defense and RNA:DNA Flipons

RPOL3 transcripts produced under stress conditions also enable another form of defense against EREs based on RNA:DNA hybrids. These RNAs serve as substrates for ERE reverse transcriptases, which use their presence to produce RNA:DNA hybrids (RDHs) that activate immune responses. Notably, cytoplasmic DRHs have been reported to be RPOL3-dependent [108]. They are not associated with the RPOL2-generated damage arising from R-loop formation [109]. They are likely generated by an active LINE-1 reverse transcriptase recruited by RPOL3 transcripts via their Poly(dA) segment, a repechage of how SINEs were first generated from 7SL RNAs. Indeed, RT inhibitors prevent the accumulation of cytoplasmic RDHs in granulocytes from patients with systemic lupus erythematosus [110]. The LINE-1 RT is efficiently primed by cytoplasmic tRNAs, short RNAs, and hairpins. The RDH produced are then thought to activate the DNA sensor cyclic GMP−AMP synthase (cGAS), leading to interferon production [111,112]. However, immunofluorescence studies do not co-localize cytoplasmic LINE-1 proteins with cGAS, but instead with the ZNA-binding protein 1 (ZBP1) sensor [108]. The findings suggest a novel mechanism in which increased RPOL3 transcription of SINEs can defend the host by triggering cell death pathways. When the execution is thwarted, either in cancer, infection, or senescent cells, chronic inflammation is instead triggered. Indeed, high levels of SINE expression characterize many of these diseases [19,113,114,115,116,117].

## 7. RPOL3 Transcripts and DNA Flipons

While RNAs can form a variety of structures and sequence-specific interactions, transcription also exploits the cycling of flipons from one conformation to another (Figure 3). Once triggered, high transcription rates drive Z-DNA formation, which the RPOL3 subunit RNAP3C likely recognizes. The interaction reinitiates assembly of the transcription complex anchored by TFIIIB, and establishes a chromatin structure permissive to high RNA expression [118].

In the human genome, pairs of RPOL3-transcribed SINEs occur in close proximity, with two-thirds being direct repeats. Around a quarter of these direct repeats are within 20 bases of each other, while ~60% are 100 or more bases apart. In contrast, around ~80% of inverted repeats are separated by at least 100 base pairs. Most direct and inverted Alu pairs share between 65% and 85% sequence identity [123]. The repeats allow RPOL3 to modulate several outcomes, depending on the local supercoiling produced as the polymerase plows through the DNA helix. The underwound DNA generated upstream of RPOL3 (indicated by “−” in Figure 3) creates a site that facilitates the wrapping of DNA around nucleosomes as they dock. The downstream positive supercoiling (indicated by “+” in Figure 3) can negate the negative supercoiling required for stable nucleosome insertion, leading to ejection of the nucleosome (Figure 3B,C).

With closely spaced direct repeats, the TUs can form an RPOL3 train, in which the downstream TU induces negative supercoiling that promotes engagement of the upstream RPOL3, thereby generating high levels f unwinding in the upstream region (Figure 3A). Readthrough of a weak termination signal or loss of the TFIIIC-binding site in the downstream TU can produce a loner RPOL3 transcript. With direct repeats farther apart, the TUs can fire independently, creating a switch that keeps only one TU active by changing nucleosome placement (Figure 3B). In one case, negative supercoiling arising just 5′ to the downstream TU can rephase a nucleosome array to mask the upstream TSS, thereby suppressing that TU. In another situation, transcriptional interference produced by readthrough of the downstream TSS by the upstream TU will disrupt readout from the downstream TU (Figure 3B). When RPOL3 TUs are convergent, the region of intense positive supercoiling can phase nucleosome arrays by displacing nucleosomes from this region, or by replacing canonical histones with those that can dock to positively supercoiled DNA, such as Centromere Protein A (CENPA) [122,124]. The underwound DNA released by the nucleosome counters the increased tension without requiring topoisomerases (Figure 3C). When RPOL3 TUs are divergent, higher levels of negative supercoiling are generated in the intervening segment (Figure 3D). If flipons are encoded in this region, the negative supercoiling generated can power the flip to an alternative conformation, such as ZNA, G-quadruplex, or a hairpin cruciform. Indeed, EREs are enriched for flipons, with both Z-DNA and G-quadruplex structures experimentally confirmed for SINEs, LINEs, and LTRs. These play roles in regulating their expression and that of surrounding genes [5,18,125,126,127,128,129].

The underwound, RNA-free regions between divergent TUs (Figure 3D) also provide landing spots for RNAs acting in trans to modulate local flipon conformations. The RNAs can dock to make triplexes. Alternatively, the process can generate R-loops, in which an RNA hybridizes to one DNA strand. The displaced DNA can then fold as a GQ or an i-motif, depending on its sequence. R-loop formation has been proposed to facilitate processes like splicing by bridging TUs with nuclear speckles [130]. Alu transcripts have also been reported to promote the assembly of enhancer–promoter condensates through sequence-specific interactions between different chromosomal regions [131]. How the production of the Alu pairs at each site is coordinately regulated remains unknown. The short length of the RPOL3 TUs makes trans sense–antisense interactions more favorable than those formed in cis. Unlike convergent RPOL2 transcripts, RPOL3 transcripts from adjacent TUs have little sequence overlap [132]. 

Collectively, these different pairings enable the chromatin remodeling necessary to mount genome-wide responses to infections induced by stress-activated RPOL3 transcription. The alternative conformations flipons adopt help maintain an open DNA state. The complexes they assemble then seed state-specific condensates necessary to protect the genome. A subset of these complexes modulate RPOL2 activity and pre-mRNA processing.

## 8. RPOL2-Produced NCRs in Host Defenses

RPOL2 transcripts play a complex role in host protection through the proteins they encode. Here, the focus is on NCRs that RPOL2 produces from EREs or targets. These include antisense RNAs, SINE dsRNAs, circular RNAs (circRNAs), and RNAs that regulate RPOL3 transcription.

### 8.1. RPOL2 and RNA Defenses Against Active Endogenous Retroelements

RPOL2 transcripts work in concert with RPOL3 to defend the host. These ancient NCRs actively target EREs expressed in the germline, where DNA is open, reflecting the nucleosome loss in sperm and the rapid cell proliferation in the embryo. They include piRNAs incorporated into a piRISC composed of PIWI proteins, which are another member of the AGO clade. In humans, short ~20-nucleotide piRNAs guide PIWIL3-containing complexes to newly evolving non-LTR retrotransposons. These short piRNAs are generated by a ping-pong mechanism, in which one piRNA directs the generation of another from a complementary RNA strand. The most highly expressed ERE strand then generates the most piRNAs. Longer ~26–30-nucleotide piRNAs are generated by phased processing, where a single strand undergoes inchworm cleavage starting at the 5′ end to generate 3′ piRNAs that target PIWIL1 to mostly LTR transcripts. The pathways can work cooperatively, with remnant RNAs from the ping-pong pathway then undergoing phased processing to yield the longer piRNAs [133]. The system is not typically expressed in normal tissues because it can potentially target host mRNAs, causing off-target effects [46,134]. DICER can also process the dsRNAs produced from RPOL2-transcribed EREs into endogenous small interfering RNAs (endo-siRNAs) [135,136,137]. This pathway later evolved into the AGO RISC loading complex (RLC) (Figure 2C,D) [53,55]. It is not thought to play a major role in normal cells, as it depends on forming long dsRNAs that can trigger interferon responses. Instead, AGO proteins bound to ERE-derived sequences have evolved other roles, particularly in early development (reviewed in [138]). Somatic cells now use KRAB-ZFP proteins to suppress transcription of both LTR and non-LTR retrotransposons [139].

### 8.2. RPOL2 Transcript Modulation of RPOL3 Transcription

RPOL2 transcripts derived from EREs also regulate RPOL3 transcripts. They target the long poly(dA) segments that drive SINE retrotransposition by capturing LINE RTs. The duplex poly(dA) tracts are susceptible to triplex formation with the NCR produced by RPOL2 docking as the third strand (Figure 1C). The poly(dA) tracts are longest in the youngest members of the Alu family, extending up to 97 nucleotides in primate-specific AluY [140]. Long noncoding RNAs (lncRNAs) exploit this vulnerability by forming triplexes that suppress SINE expression. The lncRNA KCNQ1OT1, for example, contains an extended triplet-forming poly(rA) sequence that targets SINEs genome-wide [141].

### 8.3. RPOL2 and Antisense Transcripts

Experimental evidence shows that RPOL2 can transcribe the single-stranded DNA of R-loops in the antisense direction [116], suggesting that their biology is more complicated than first imagined. Previously, ~20% of human genes were identified as having overlapping antisense transcripts [142]. Convergent transcription was thought to produce transcriptional interference, providing a means for regulating gene expression [143,144,145]. A recent study has confirmed the high frequency of convergent transcription at nearby promoters, with a median separation of 413 bp (range: 2 to 2301 bp). These promoters did not exhibit transcriptional interference [132]. Instead, a coordinating command center (CCC) that lies between them synchronizes promoter activity. The CCC prevents clashes between the RPOL2 polymerases transcribing each TU by gating their activity (Figure 4A). The design also enables regulation via small RNAs that target a specific transcript, thereby suppressing or activating a particular promoter. The RISCs formed can also cleave nascent transcripts, leading to the dynamic ejection of RPOL2 [146]. Both NCR and CCC sequences can undergo selection to optimize cell survival and phenotype. These NCRs can also modulate alternative flipon folds, as previously proposed [147], including GQs, intercalated cytosine-rich i-motifs, hairpins, ZNA, and slipped-strand structures [5,148,149]. Notably, CCCs between convergent promoters show an increased propensity to form both GQs and R-loops [132]. In addition, lncRNAs, including those derived from ERE, can also form triplexes (as extensively reviewed in [150]). 

### 8.4. RPOL2 and SINE Transcripts

Alu inverted repeats (AIRs) transcribed by RPOL2 within the same TU can also regulate immune responses by forming dsRNAs (Figure 5A). Viruses also produce immunogenic dsRNAs, which are countered by defenses evolved against EREs [129,151,152,153,154,155]. Many reviews explore how dsRNAs impact RNA stability, render miRNA target sites inaccessible, and serve as substrates for the RNA-editing enzymes double-strand-specific adenosine-to-inosine deaminases ADAR1 (encoded by *ADAR*) and ADAR2 (encoded by *ADARB2*) [156,157,158] (Figure 5B). Here, the focus is on how ERE-generated dsRNAs now encode a universal host-based defense [118,129]. The design contrasts with that found in classic genetic organisms like flies and roundworms. In these organisms, RNA interference pathways specifically target pathogen sequences. RTs are required to amplify responses. Hosts under retrotransposition attack select against such enzymes, as they favor the attacker rather than the defender [118]. Notably, the piRNA amplification schemes do not depend on such enzymes. In contrast to the pathogen-specific RNA defenses of flies and worms, the host-based defenses do not depend on RTs to stop invasive threats. 

In the other case, host antiviral defenses are activated by viral transcription factors (TFs). The strategy fails when pathogens can mutate the TFs or their motifs faster than a host can mirror the changes. The host must instead control threat detection and response independently of pathogen sequence variations. It also potentially fails when host TF motifs used in host-based defenses arise from ERE sequences. The TFs driving the response also activate transposon expression [159]. This holds for EREs like SINEs, which do not encode proteins and instead depend on host-encoded factors for expression. RNA- and DNA-based sequence-specific defenses are further complicated by the incorporation of ERE sequences into host genes, making it harder to distinguish host and ERE-derived RNAs. An elegant solution is provided by flipons, which direct responses by their structure rather than their sequence [1,87]. In short, the host uses the flipons within EREs to regulate the host defensive response. The level of Z-RNAs generated determines the outcome. Figure 5 and Figure 6 illustrate the design.

**Figure 5:**
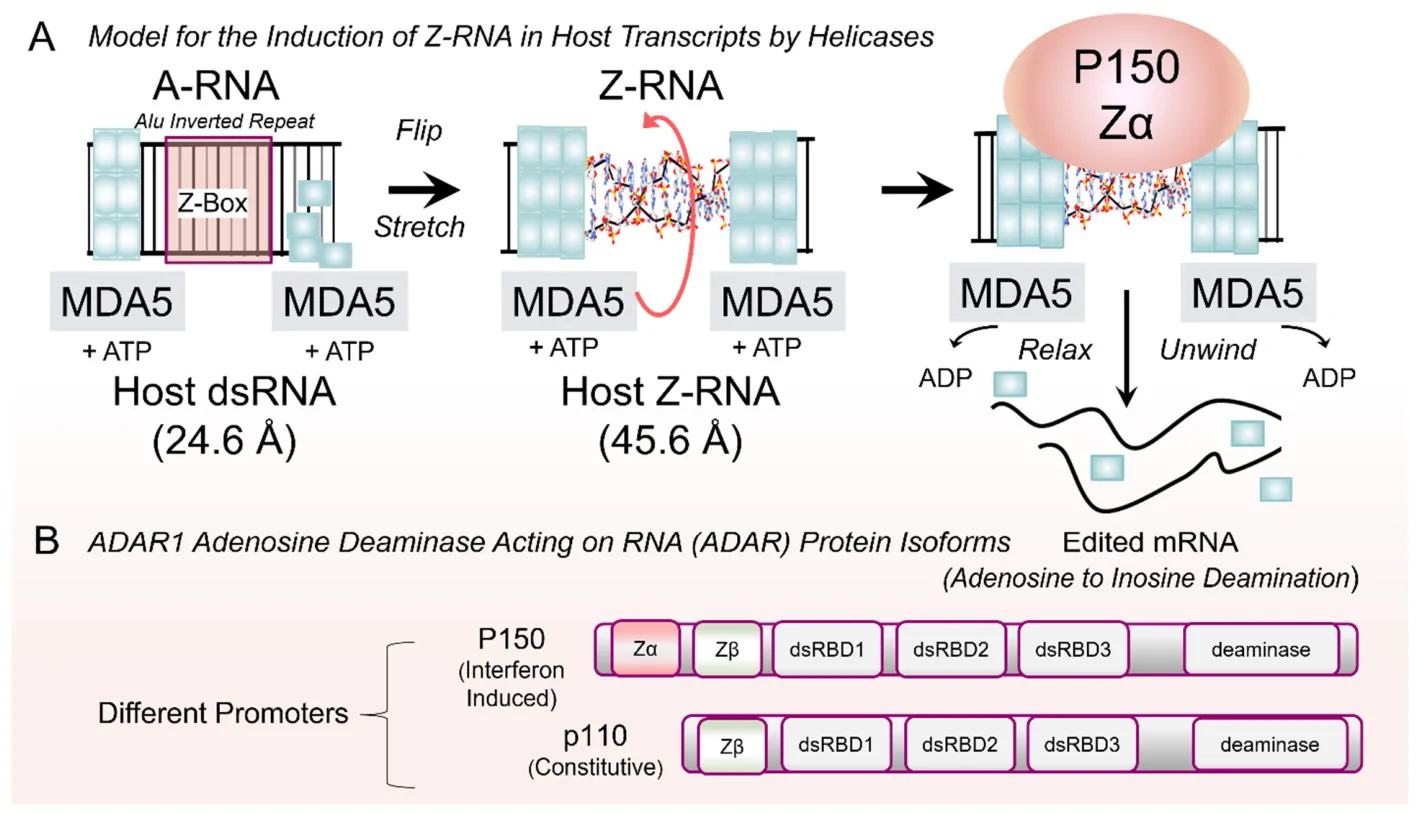
Helicases induce the flip to Z-RNA by tensioning dsRNA. (**A**) The Melanoma differentiation-associated protein 5 (MDA5) protein (encoded by the interferon-induced helicase C domain-containing protein 1 [IFIH1] gene) assembles on dsRNA stochastically and forms a filament that scaffolds the interferon response. During the process, the dsRNA is tensioned. This stretch is sufficient to flip an Alu inverted-repeat sequence to Z-RNA, as it contains a conserved Z-prone sequence called a Z-Box. In one model, the transition relaxes the filament, causing the MDA5 filament to collapse, with ATP hydrolysis completing the cycle. Z-RNA formation engages double-stranded RNA-specific adenosine deaminase (ADAR1) p150 through its Zα domains. The enzyme then deaminates adenosine to form inosine, promoting disposal of the dsRNA and terminating interferon induction by this substrate. (**B**) ADAR1 has two isoforms. Only the interferon-induced p150 isoform recognizes Z-RNA through the Zα domain. Zβ does not recognize Z-RNA but, like Zα, will bind GQ with high affinity. The p110 isoform is constitutively expressed and mostly nuclear, whereas p150 is mainly cytoplasmic. (Adapted from [160]).

**Figure 6:**
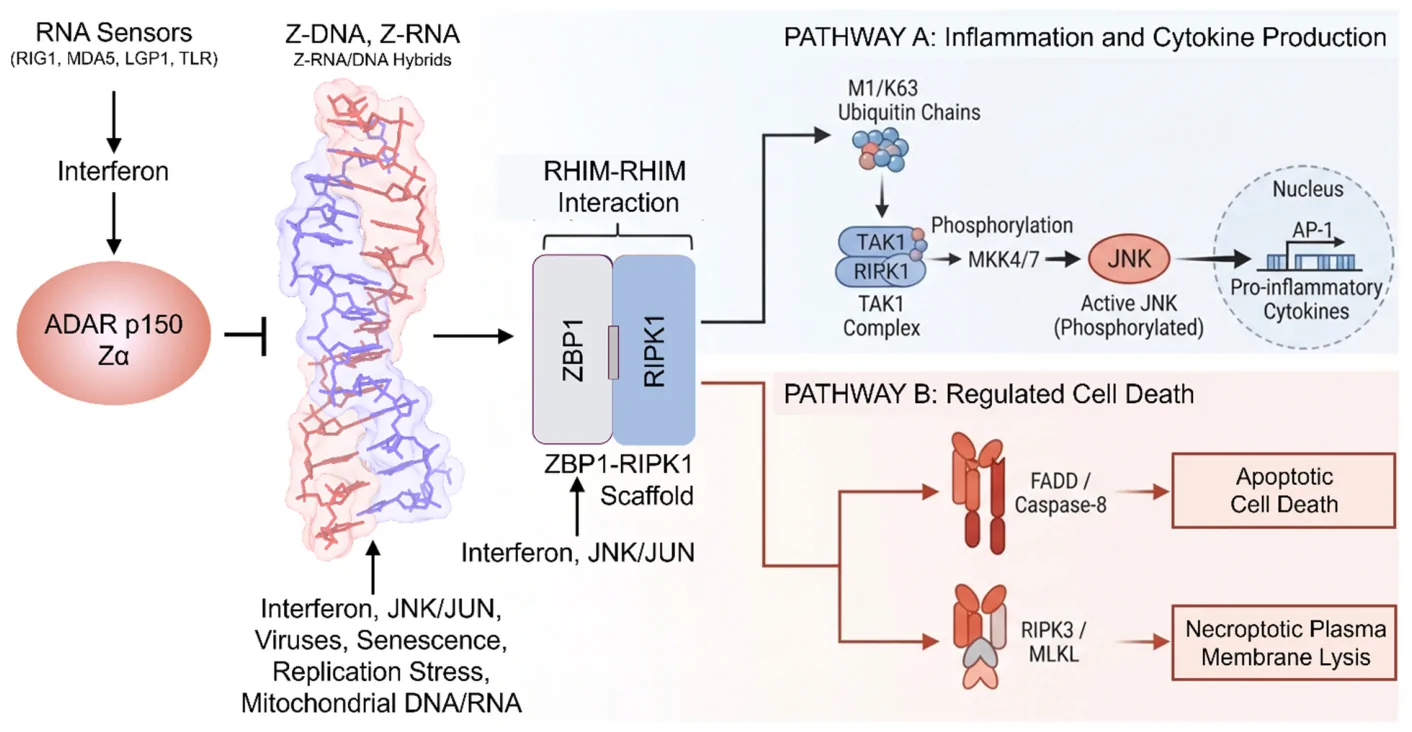
Double-stranded RNA-specific adenosine deaminase (ADAR1) p150 negatively regulates ZNA binding protein (ZBP1) activation. ADAR1 p150 is induced when RNA sensors initiate interferon production. In humans, ZBP1 signals through receptor-interacting serine/threonine-protein kinase 1 (RIPK1) to activate downstream cytokine responses and induce cell death via apoptosis or necroptosis. The pathway activated is context-specific. Interferon strongly induces ZBP1 during stress and infection, to levels that exceed the capacity of ADAR1 p150 to prevent its activation and thereby silence the responses it induces.

It is based on AIRs inserted into normal host transcripts. The default host response to these dsRNAs is to stochastically assemble filaments of melanoma differentiation-associated protein 5 (MDA5, encoded by the interferon-induced helicase C domain-containing protein 1 *IFIH1* gene) [161]. If left unchecked, the filaments activate an interferon response [162,163]. However, those host-encoded dsRNAs derived from SINEs incorporate flipons. They contain Z-boxes that flip to Z-RNA when the filament is stretched (from 24.6 to 45.6 Å). One model suggests that the longer Z-RNA relaxes the tension and triggers MDA5 to dissociate and hydrolyze ATP (Figure 5A) [7,18,160]. The Z-RNA then localizes the p150 isoform of ADAR1 to the dsRNA via its Z-RNA-specific Zα domain (Figure 5B). Subsequent deamination of the dsRNA prevents further MDA5 filament formation, thereby preventing an immune response against self. Inosine-specific RNases then clear the modified RNAs. Interferon induces the p150 isoform as part of the initial response. In the absence of interferon, expression of an ADAR1 p110 isoform is driven from a different promoter (Figure 5B) [164].

Genetic proof that ADAR1 p150 suppresses interferon activation comes from individuals from families that express p110 normally but transmit one p150 allele with either an N173S or a P193A loss-of-function variant that no longer binds ZNA, combined with a p150 null allele. Because a normal allele no longer masks the loss-of-function allele, the variant maps directly to the phenotype. The individuals with the unmasked loss-of-function haplotype develop an Aicardi–Goutières type 6 interferonopathy [165].

AIRs inserted into normal host transcripts allow these RNAs to be distinguished from viral RNAs, which lack this feature. Furthermore, the many insertions into normal host messages make it difficult for the virus to suppress each one individually. Viruses such as vaccinia have a Zα ortholog that directly binds Z-RNA, thereby masking it [166]. Viruses like influenza and herpes simplex try to prevent host responses by trapping host polymerases on host genome DNA. Their proteins disrupt the normal termination of host transcription, causing the host polymerase to continue elongating along the chromosome. This process shuts down host RNA production. The host counters this by enriching Z-RNA-forming EREs just past the normal transcription termination site. High ERE levels are expressed when viral proteins disrupt termination, causing Z-RNA to accumulate as well. The volume of Z-RNAs produced overwhelms ADAR1 p150’s ability to squelch them [127,129,167,168,169].

The Z-RNAs then activate ZBP1, the only other Zα-containing protein in the human genome. This protein is also interferon-induced and often expressed at much higher levels than ADAR p150. ZBP1, along with Z-RNA-forming transcripts, is also induced by the Janus kinase (JNK) signaling pathway [170,171] (Figure 6). ZBP1 can then trigger scaffold assembly through its receptor-interacting protein homotypic interaction motifs (RHIMs) [172,173,174]. Depending on the cell context, the RIPK1-dependent processes in humans can lead to an inflammatory form of cell death called necroptosis, a silent apoptotic cell death, or a chronic inflammatory response [175]. Other downstream processes triggered by ADAR1 p150 loss also have adverse outcomes. The neurological damage in mice with an Aicardi–Goutières syndrome-like phenotype caused by a strongly penetrant loss-of-function W195A Zα allele [176] is not rescued by deletion of either ZBP1 or the dsRNA Protein Kinase R (PKR) sensor that induces interferon via MDA5.

The host-based strategy depends on multiple sensors of a cell’s well-being that activate interferon responses. These sensors warn of an imminent threat by detecting bacterial metabolites, viral transcripts, cytoplasmic DNA, and host DNA damage. They initiate a host-based defensive response. Both interferon and stress kinase pathways are triggered. These pathways also induce transcription of EREs that are otherwise suppressed in normal cells [129,177,178,179]. The dsRNAs formed further stimulate the interferon response. The outcome depends on how much Z-RNA accumulates. If Z-RNA levels exceed the ADAR p150 threshold, the response amplifies to eliminate the threat.

## 9. Circular RNAs and ADAR1 p150

SINEs can also generate circular RNAs (circRNAs) through aberrant splicing of AIRs (Figure 7). The circRNAs are formed by back-splicing of a downstream splice donor to an upstream splice acceptor site. Back-splicing is promoted by Alus with complementary sequences. They bring the splice sites into close proximity by hybridizing to form loops. A subset of circRNAs is exported to the cytoplasm. Many contain repeat sequences complementary to the UTR sequences of cytoplasmic mRNA (Figure 7C) [180,181]. When they form hybrids, the circRNAs can activate Staufen 1 (STAU1)-mediated messenger RNA decay (SMD) [180]. In other cases, circRNAs incorporate exons and modulate the stability of mRNAs that have complementary exons. Several such exon–exon pairs have been identified. These circRNAs often retain exon-junction complexes that induce the nonsense-mediated decay of the mRNAs they bind. The interactions can prevent the translation of cellular proteins necessary for survival [181]. Examples include the circRNAs: hsa_circ_0002082 and circ hsa_circ_0008496 that downregulate the pro-apoptotic BIM protein (Bcl-2-like protein 11 encoded by BCL2L11) [181]. Another set of circRNAs protects cells against inflammation. They retain sequences that form 12–16 bp dsRNA. These stems are not long enough to activate interferon responses but instead inhibit PKR-mediated inflammatory responses [182] (Figure 7B). A different set of circRNAs is reported to inhibit cyclic GMP−AMP synthase (cGAS) activation of the interferon response [183]. Overall, these mechanisms are anti-inflammatory.

Back-splicing is promoted by processes that cause RPOL2 pausing and serve as sensors for oxidative DNA damage and replication stress, both of which can induce Z-DNA and GQ (Figure 7A) [184,185,186,187,188,189]. Loss of either ADAR1 p150, which engages both conformations, or the helicase ATP-dependent RNA helicase A (RHA encoded by DHX9) greatly increases accumulation of circRNA and of the dsRNAs formed by circRNA hybrids. Interferon-induced RNase L cleaves the single-stranded circRNA to relieve their suppressive effects, but not the dsRNA hybrids they form. Subsequent cell death can be triggered through several cell death pathways besides ZBP1 [181,190,191] (Figure 7D). This circRNA accumulation is proposed to exacerbate several neurodegenerative diseases [191] and may explain the neurological phenotype of the loss-of-function ADAR1 p150 W195A Zα allele [179].

## 10. RNA Polymerase Pausing, Oxidative Damage and DNA Repair

Oxidative DNA damage in tissues with high mitochondrial activity, such as the nervous system, contributes to circRNA formation by enhancing polymerase pausing, allowing more time for Alu RNA hybrids to form and for back-splicing to occur. Increased levels of reactive oxygen species (ROS) promote ZNA formation by oxidizing guanosines. These adducts impair B-DNA reformation by Z-flipons during the transcription cycle [184,185,186,187,188]. The repair enzyme 8-oxoguanine DNA glycosylase (OGG1) cannot excise the oxidized base in the Z-DNA conformation. In this situation, localization of the DHX9 helicase by ZNA-bound ADAR1 p150 may be essential for recruiting the repair complex (Figure 7). Subsequently, DHX9’s interaction with poly[ADP-ribose] polymerase 1 (PARP1) recruits OGG1 to the lesion. Z-DNA thereby facilitates the rapid genome scanning needed to localize OGG1 to lesions in active genes [192,193].

### 10.1. Mitochondrial DNA-Activated Immunity

In addition to causing oxidative damage, damaged mitochondria release their oxidized nucleic acids into the cytoplasm, also leading to ZBP1-dependent outcomes. Notably, ZBP1 can enhance cGAS activity by docking to Z-DNA formed within the covalently closed circular oxidized mitochondrial genome, while engaging the N-terminus of cGAS through its RHIM domains. Interferon induced by 2′3′-cGAMP-mediated activation of the stimulator of interferon genes (STING) then further increases ZBP1 expression, creating a positive feedback loop that promotes cell death [187] (Figure 6). 2′3′-cGAMP also activates STING and induces ADAR1-mediated autophagy in bystander cells, promoting their death after interferon-induced upregulation of ZBP1, as seen in SARS-CoV-2 infection [194,195,196,197]. Cell death is further enhanced by the DNA double-strand break sensor Meiotic recombination 11 (MRE11) during DNA damage repair. The enzyme releases STING from the acidic nucleosome patch that sequesters the protein in an inactive state [198]. ZBP1 potentially promotes this release by directly interacting with MRE11 and inhibiting its repair activity [199]. The cGAS-ZBPI interaction can also be triggered by extracellular neutrophil nets that play a role in acute inflammatory pancreatitis and harbor both Z- and G-flipons [200], as well as in systemic lupus erythematosus [201].

### 10.2. RNA Polymerase Pausing and Replicative DNA Damage

Persistent Z-DNA from oxidative damage inhibits polymerase elongation, increasing negative supercoiling as trailing polymerases arrive, stall, and eject from the DNA. This process generates high levels of positive supercoiling ahead of the lesion, destabilizing chromatin and causing extensive upstream DNA unwinding. The 5′ negative supercoiling promotes R-loop formation, in which the displaced single-stranded DNA is particularly prone to further oxidative damage, as well as the flip to Z-DNA and the folding of GQ by both RNA and DNA. The repair complexes recruited further exacerbate the stalling of other trailing RNA polymerases by these alternative structures [185]. If unresolved, this process leads to DNA damage, particularly during cell division, when collisions between RNA and DNA polymerase at stalled replication forks amplify the negative outcomes. These collisions are frequently associated with RPOL3 stalls at inverted repeats [202].

Replicative stress also results in cytoplasmic DNA fragments and the accumulation of sub-telomeric RNAs that can activate ZBP1 [203]. These outcomes are augmented by the recent discovery that both the Zα domains of ADAR1 and ZBP1 can dock to both GQ DNA and RNA, with the GQ terra RNA activating ZBP1 [88,89,204,205]. ADAR1 Zβ (Figure 7) also has a strong preference for telomeric GQ [89]. Previously, it was known that Zβ does not bind Z-DNA or Z-RNA because the structure-specific tyrosine residue in Zα is replaced by isoleucine in Zβ. Unlike Z-RNA, which forms from dsRNA, GQ can form in ssRNA. Potentially, ADAR1 p150 is optimized to bind Z-RNA formed in the cytoplasm, while Zβ recognizes the GQ formed by Alu elements in pre-mRNAs and can offset the risks they pose associated with mis-splicing or R-loop-induced DNA damage (Figure 8) [88].

## 11. Emerging Themes

The protection of hosts by the mechanism described here and summarized in Table 1 represents a series of ad hoc adaptations that enabled hosts to survive and transmit their genomes to progeny. While we tend to look for well-engineered systems and imagine Nature can likewise order its catalog, reality is far different. As the separate evolution of the AMC and RLC machinery shows, solutions often overlap, creating redundancy and resilience. Retrotransposons are both targeted and exploited, with some adaptations limiting ERE spread in a sequence-dependent manner. At the same time, the alternative folds they adopt, such as ZNA, G-quadruplexes, and triplexes, undergo exaptation to program cell responses. Of these structures, ZNA sensors enable host-encoded immune defenses that do not depend on reverse transcriptases for amplification while protecting against a wide range of pathogens [118,129].

This regulation of pathways by alternative structures dates back to the early eukaryotic progenitors, and likely preceded the use of sequence-specific TFs. These structures also enabled condensate assembly that defined spaces for different chemistries to evolve, including RNA catalysts, while protecting their interior from external hazards [206]. The structures formed depend on the flipon motif involved. As extensive studies of Z-DNA show, the transition to an alternative fold can depend on salt, metal, temperature, and pH conditions [207]. Sequence-specific targeting of the motifs provides another way to modulate their conformation. Nature likely exploited nucleic acids to control flipon conformation early on in protocells. Trial and error improved on earlier iterations. Sequence-specific proteins arrived later, building on proven RNA adaptations, as evidenced by small RNAs that now guide protein effectors and catalyze protein synthesis in the ribosome. The protein tools evolved were more robust and came with modular domains that could be assembled, duplicated, or otherwise modified to improve a species’ survival and reproductive success. A key imperative was to protect the genome from invasive replicants.

Eukaryotic RNA polymerases have adapted to defend the host. The three human polymerases stem from the same ancestral enzyme. Out of the 14 subunits in RPOL1, 12 subunits in RPOL2, and 17 subunits in RPOL3, five core subunits (RPB5, RPB6, RPB8, RPB10, and RPB12) are completely identical and physically shared between them [208]. Yet each protects cells against existential threats differently. RPOL3 transcribes SINEs to enable defenses based on small NCRs that regulate mRNA expression, translation, and protein function in normal cells. When stressed, RPOL3-dependent SINE transcription shifts cells from a relatively quiescent state to one primed to defend itself. In this process, RPOL3 modulates RPOL2 transcription, selectively activating pathways that robustly repel threats from within.

ERE-derived sequences regulate RPOL2 transcription during development [209]. NCRs can also target these elements to regulate chromatin conformation and the flipon structures that modulate gene expression. These pathways so far have proven challenging to exploit therapeutically. Explicitly targeting regulatory flipons offers a new opportunity.

## Figures and Tables

**Figure 2:**
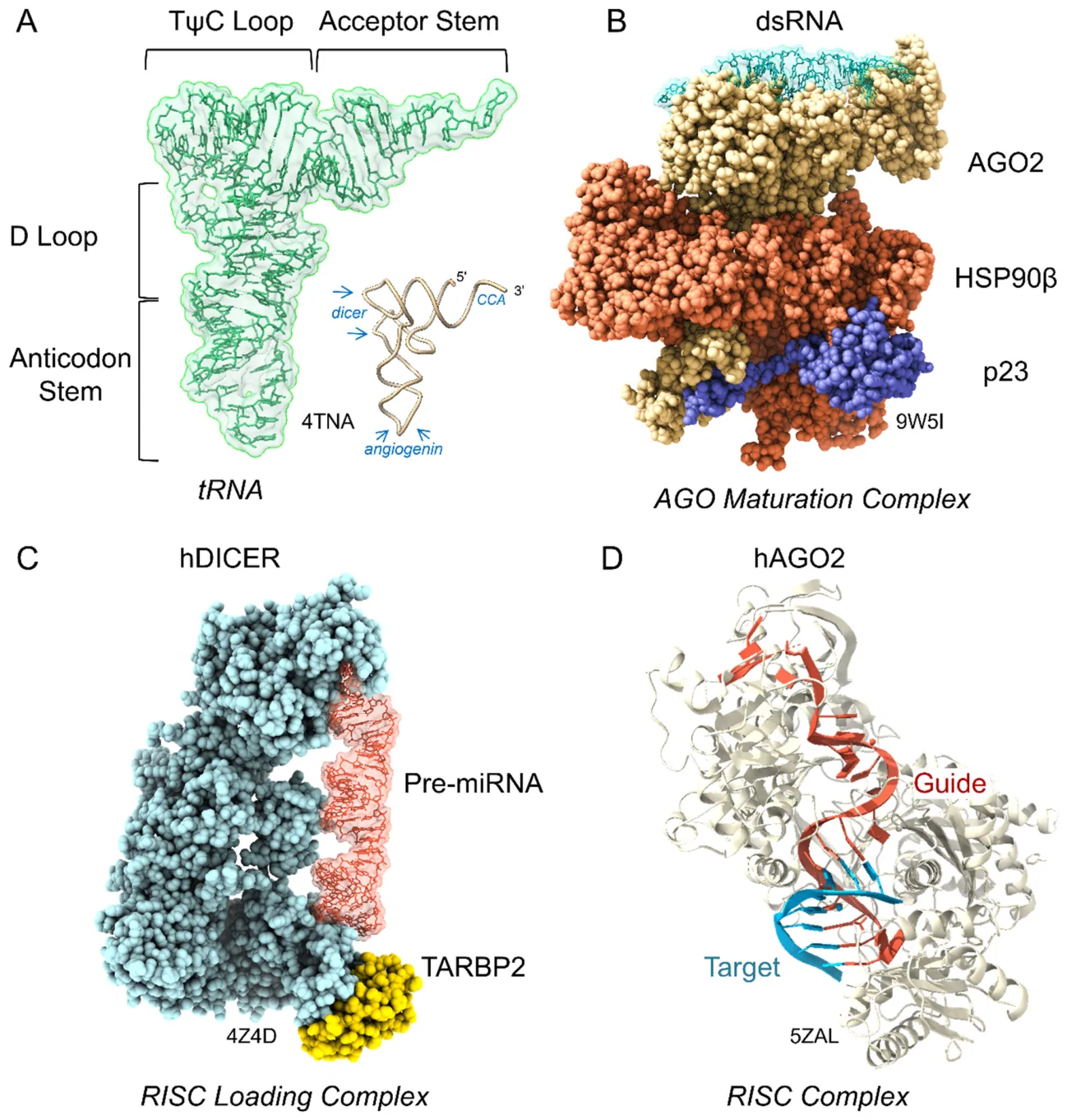
The L-shaped tRNA structure that is processed to generate fragments loaded onto Argonaute (AGO) by the AGO maturation complex (AMC) and the RNA-induced silencing complex (RISC) loading complex (RLC). (**A**) The tRNA L-shaped fold (4YNA). The TψC-acceptor double-stranded RNA (dsRNA) duplex is long enough to engage the AMC, but the D-loop anticodon-stem duplex is too short. The inset shows the tRNA backbone with cleavage sites for Dicer and Angiogenin. (**B**) The human AMC loaded with the let-7a-1 miRNA (9W51). All coordinates were obtained from the Protein Data Bank [45]. (**C**) The human RLC is composed of Dicer and the TAR RNA-binding protein 2 (TARBP2) with a pre-miRNA loaded. (**D**) The RISC with the guide strand (fuchsia) bound to a target RNA (teal).

**Figure 3:**
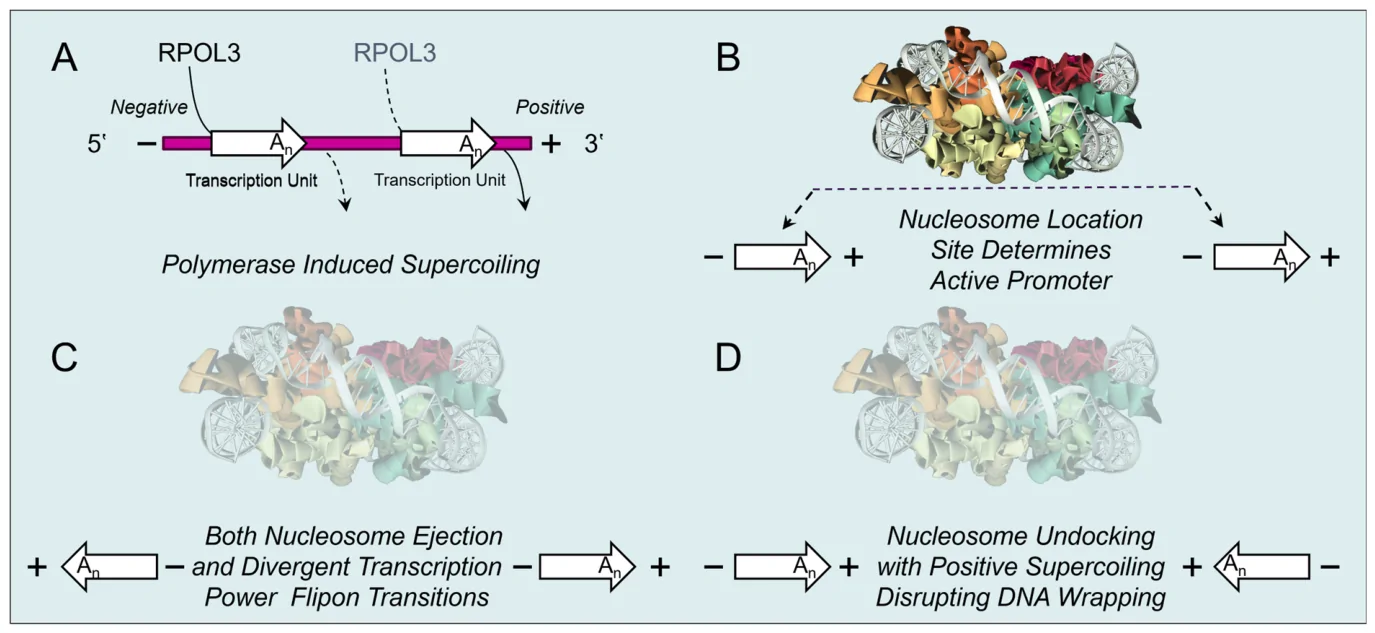
The potential for RNA Polymerase 3 (RPOL3)-transcribed short interspersed nuclear elements (SINE) repeats to regulate nucleosome placement, transcription rate, and flipon conformation. The arrows indicate the direction of transcription, and the “−” and “+” signs represent regions of negative and positive supercoiling generated by RPOL3 according to the twin domain model of Liu and Wang [119]. The “A_n_” indicates the variable Alu poly(dA) 3′ tail present just before the oligo-dT transcription termination site. An Alu is a SINE family member characterized by the fragment sizes produced by digestion with the *AluI* restriction enzyme. Nucleosomes are shown wrapped in negatively supercoiled DNA. (**A**) Closely spaced direct repeats can be transcribed by two polymerases acting in tandem to increase upstream local negative supercoiling, or by a single RPOL3 if the oligo-dT termination signal on the first is weak, or the downstream transcription (TFIIIC) binding site has been lost. (**B**) Direct repeats spaced further apart can influence the site of nucleosome insertion. The upstream transcription unit (TU) can generate negative supercoiling to promote nucleosome insertion upstream of that element, and positive supercoiling to displace the nucleosome downstream. Alternatively, the downstream TU can unwind the DNA immediately upstream to promote nucleosome docking between the two TUs. This nucleosome phases the upstream nucleosome array. Nucleosome placement over the upstream TU promoter then prevents RPOL3 from docking and transcribing this region. (**C**) Divergent transcription of inverted repeats, or nucleosome ejection, greatly increases local negative supercoiling between the TUs. The energy stored in the negative supercoils then powers the transition of promoter flipons to an alternative state. The formation of Z-DNA and G-quadruplexes then helps localize transcription and RNA processing complexes to these sites [120,121]. (**D**) Convergent transcription of inverted repeats promotes nucleosome undocking by increasing positive supercoiling, promoting replacement of canonical histones with others, such as Centromere Protein A (CENP-A), that structure chromatin differently [122].

**Figure 4:**
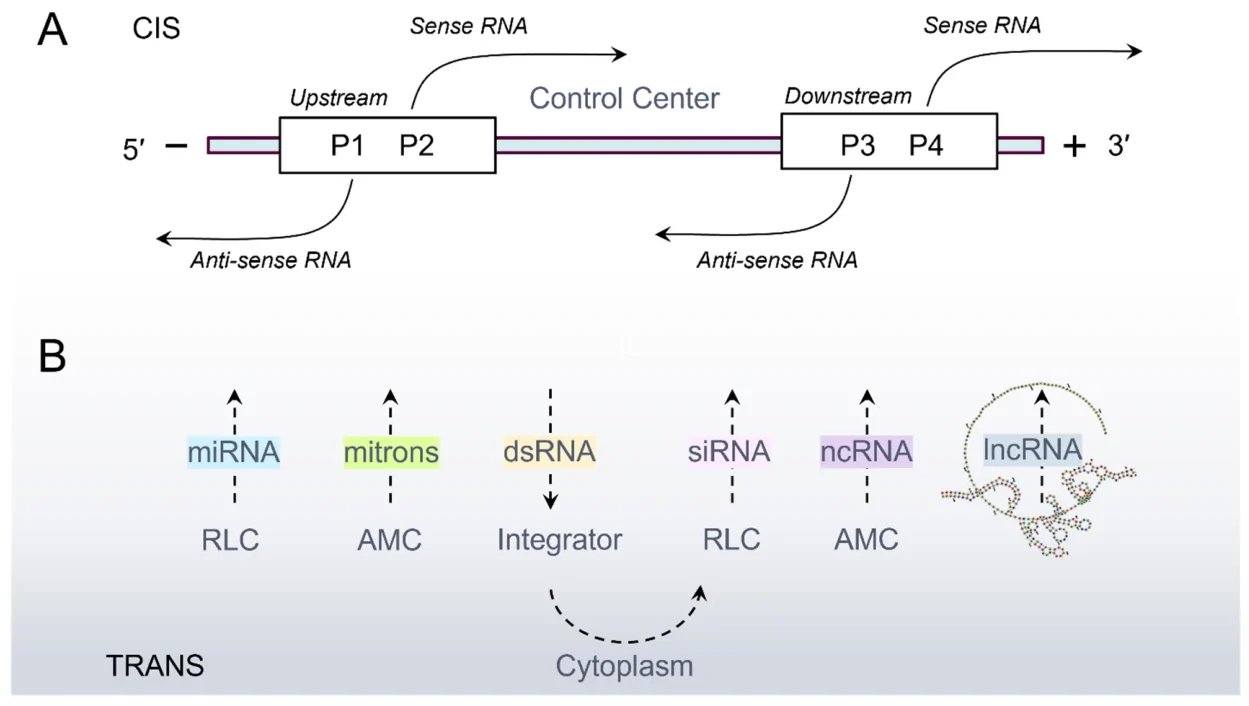
Promiscuous gene transcription modulated by noncoding RNAs (NCRs) acting in cis and in trans. (**A**) Cis regulation of gene expression. Recent evidence reveals that most promoters undergo bidirectional transcription, with ~20% converging with another promoter. The coordinating command center (CCC) lies between them, ensuring that the transcription of each TU is timed to avoid collisions between RNA polymerase 2 (RPOL2) in normal cells. (**B**) Trans regulation of gene expression. Trans-acting small RNAs and lncRNAs target the transcripts and modulate their production. When the timing is disrupted during stress, antisense transcripts may arise. The dsRNAs formed are captured by the integrator complex and passed into the RNA-induced silencing complex (RISC) pathway. Other processing pathways also produce RISCs with guides processed from miRNAs and from other NCRs, such as tRNAs and other RNA polymerase 3 (RPOL3) transcripts. RNA guides can modulate flipon conformation through direct interactions with DNA that promote or prevent conformational change, or indirectly through the negative supercoiling generated by processive enzymes such as polymerases and helicases.

**Figure 7:**
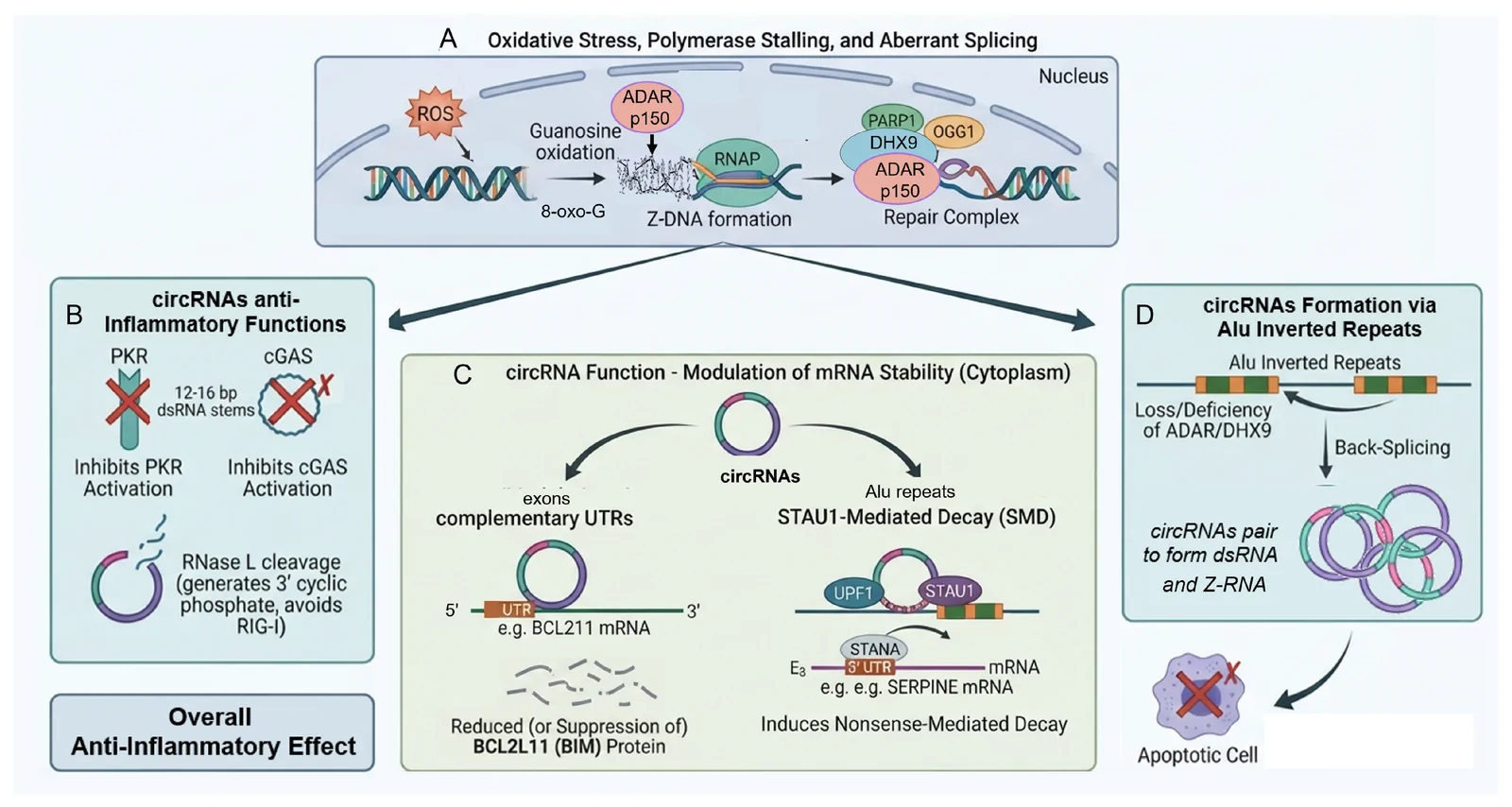
Double-stranded RNA-specific adenosine deaminase (ADAR1) p150 can also suppress back-splicing of RNA promoted by Alu sequences near splice junctions. When folded into dsRNA, Alu sequences can pair downstream splice donors with upstream splice acceptors to yield circular RNAs (circRNAs). (**A**) Back-splicing is promoted by RNA pausing that can result from oxidative DNA damage or the alternative flipon structures and repair complexes that result. (**B**) The circRNAs contain short dsRNAs that can inhibit Protein Kinase R (PKR) and cyclic GMP−AMP synthase (cGAS) activation, preventing interferon activation. (**C**) The circRNA sequences can promote the triage of pro-apoptotic messages by pairing with sequences in their 3′ UTRs and activating RNA decay pathways. (**D**) ADAR1 p150 and DEAH box protein 9 (DHX9) deficiency leads to the accumulation of circRNAs that pair to form dsRNA tangles that trigger massive cell death.

**Figure 8:**
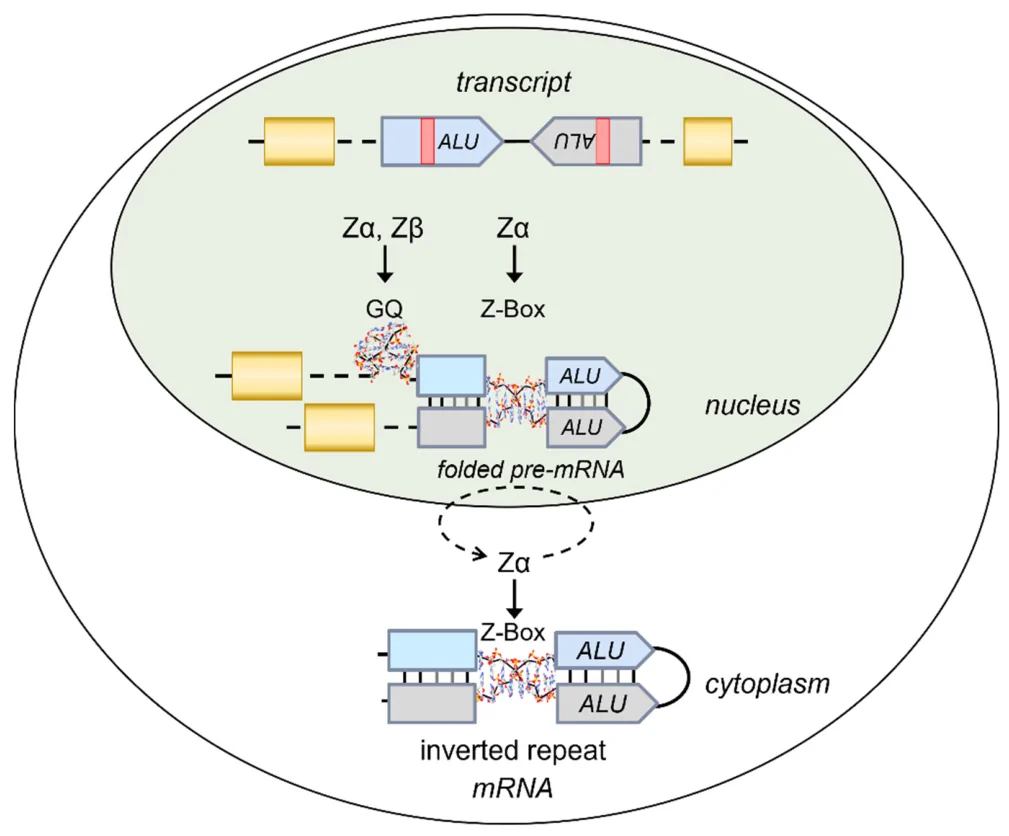
Double-stranded RNA-specific adenosine deaminase (ADAR1) p150 and p110 isoforms modulate distinct cellular outcomes. The Zα domain can localize ADAR1 to Z-RNA formed by Alu inverted-repeat dsRNA and promote editing of that substrate, thereby turning off interferon responses against self-RNAs and preventing ZBP1 activation. The Zα domain can also localize the enzyme to non-host Z-DNA or Z-RNA, disrupting viral transcript processing and translation through editing and dsRNA binding. The ADAR1 Zβ domain in the nucleus can disrupt ssRNA Alu transcripts by binding to the G-quadruplexes (GQ) they form. This inhibits the mis-splicing and DNA damage they produce (adapted from [89]).

**Table 1:** A myriad of different ncRNA pathways protect the host against pathogens and itself.

Noncoding RNA Type	Transcribing Polymerase	Associated Pathways & Complexes	Defense Against Retrotransposons & Pathogens	Protection Against Autoimmunity
tRNAs & tRNA fragments (tRFs), tRNA halves, and quarters)	RNA Polymerase III (RPOL3)	Argonaute maturation complex (AMC) (HSP90β, AGO1/3/4, p23 cochaperone); RISC loading complex (RLC) (Dicer, TARBP2, PACT)	LTR retrotransposon blocking: LTR retrotransposons rely on host tRNAs to prime reverse transcription. A specific 18 bp tRNA fragment (lacking the 3′ CCA tail) binds directly to primer binding sites to inhibit LTR retrotransposition. Viral gene-silencing modulation: tRF5^GluCTC^ is strongly induced during respiratory syncytial virus (RSV) infection to regulate silencing.	Transcript silencing & homeostasis: AMC loads duplex tRFs onto Argonaute during normal cell states to suppress immunogenic or stress-induced transcripts. Dimer masking: tRNA halves (e.g., tRNA^Gly^, tRNA^Glu^) dimerize to mask specific sequences, preventing excitotoxic neuronal necrosis and age-associated mitochondrial translation disruption. La protein protection: La binds pre-tRNA 3′ ends to prevent aberrant loading onto RNA silencing machinery.
SINEs & Alu elements (including Alu inverted repeats—AIRs)	RNA Polymerase III (RPOL3) for single-copy SINEs RNA Polymerase II (RPOL2) for gene-integrated inverted repeats	MDA5 filament assembly; ADAR1 p150-Zα editing complex EDC4-XRN1 decay pathway cGAS-STING & ZBP1-RIPK1 death pathways	Pathogen-induced interferon: Stress (viral infection, heat shock) induces RPOL3 Alu transcription, producing uncapped triphosphate RNAs that activate RIG-I-mediated interferon responses. RT hijacking: Alu SINE transcripts act as substrates for active LINE-1 reverse transcriptase to form RNA:DNA hybrids (RDHs), activating cGAS and ZBP1 to destroy infected/dysfunctional cells. Pathogen-induced cell death: Viruses that disrupt transcript splicing or termination cause massive accumulation of Z-RNA, forming retrotransposon sequences, triggering ZBP1-RIPK1-driven cell death to arrest viral replication.	ADAR1 p150 silencing of interferonopathy: Folded AIR dsRNAs form “Z-boxes” that transition to Z-RNA when stretched, collapsing immunogenic MDA5 filaments. The ADAR1 p150 isoform binds Z-RNA via its Zα domain and performs A-to-I editing, preventing chronic interferon activation and severe autoimmunity (e.g., Aicardi–Goutières syndrome). End masking: RPOL3 transcripts are masked at their 5′ ends by SRP9/14 (preventing RIG-I binding) and shielded at their 3′ ends by the La protein to preserve self-tolerance. SUV39H1 silencing: Chromatin marking by SUV39H1 suppresses RPOL3 SINE transcription in normal cells to avoid retrotransposition and inflammatory stress.
Enhancer RNAs (eRNAs) RPOL3 regulation of RPOL2	RNA Polymerase III (RPOL3) (functioning to modulate RPOL2 activities)	RPOL3 activation of RPOL2 enhancers EDC4-XRN1 decapping and decay pathway RPOL2 transcription pre-initiation complex blockade	Enhancer-mediated induction: Under stress or through physiological cues (e.g., retinoic acid induction), localized RPOL3 transcription of specific Alu elements promotes RPOL2 transcription of nearby survival genes. Transcriptional blockade under stress: During cellular stress (heat shock or adenovirus/HSV-1 infection), rapid RPOL3 transcription produces high-affinity Alu RNAs that structurally mimic the RPOL2 elongation complex. These Alu transcripts bind directly to RPOL2, disrupt its contacts with promoter DNA, halt normal transcription, and prevent ectopic activation of developmental endogenous retroelements (EREs). Antiviral interferon induction. Stress-induced RPOL3 transcription yields uncapped triphosphate RNAs that trigger the RIG-I-directed interferon response to defend the cell.	Post-transcriptional decay loop: To prevent over-accumulation, exported RPOL3 Alu transcripts bind to AGO3. This complex scaffolds the EDC4 (RCD8) decapping complex and recruits XRN1 to degrade complementary 3′ UTRs of critical mRNAs (like TDGF1 and NANOG). RPOL3 increases RPOL2 transcript levels, while later translation inhibition helps maintain cell homeostasis by regulating protein levels.
piRNAs (Piwi-Interacting RNAs)	RNA Polymerase II (RPOL2)	piRISC complexes involving PIWI clade proteins (PIWIL1, PIWIL3); utilizes the ping-pong mechanism and phase processing	Germline genome defense: Silences active LTR and newly evolving non-LTR retrotransposons in germline tissue to protect the host genome across generations.	Somatic restriction: Because piRNAs can target a vast range of transcripts and cause toxic off-target autoimmune damage, this pathway is strictly restricted and not typically expressed in normal somatic tissues. Somatic tissues instead deploy the sequence-specific KRAB-ZFP family during development to safely suppress retroelements.
Endogenous siRNAs (Endo-siRNAs)	RNA Polymerase II (RPOL2)	Dicer-dependent pathway feeding into the RISC loading complex (RLC)	Somatic ERE silencing: Targets and suppresses non-LTR LINE and SINE retrotransposons in somatic tissue by cutting the double-stranded RNAs (dsRNAs) they produce.	Immunogenic dsRNA clearance: Keeps ERE-derived dsRNAs cleared. Constant degradation of these duplexes prevents the accumulation of immunogenic dsRNA tangles that would otherwise chronically activate MDA5 or PKR sensors, avoiding systemic inflammation and self-attack.
Circular RNAs (circRNAs)	RNA Polymerase II (RPOL2) (via Alu-driven back-splicing of pre-mRNAs)	Back-splicing pathway regulated by ADAR1 p150 and DHX9 helicase	Dysfunctional cell elimination: Under stress or ADAR1 p150/DHX9 deficiency, the massive accumulation of circRNA dsRNA tangles triggers cell death. Triage of messages: circRNA sequences pair with the 3′ UTRs of pro-apoptotic mRNAs, activating decay pathways to regulate survival programs.	PKR and cGAS inhibition: circRNAs contain short stems (12–16 bp dsRNA) that are too short to trigger interferon but bind and inhibit PKR-mediated inflammatory responses and cGAS activation. Avoiding RIG-I: When cleaved by RNase L, circRNAs yield 3′ cyclic phosphates rather than immunogenic 5′ phosphates, preventing accidental RIG-I activation.

## Data Availability

No new data were created or analyzed in this study. Data sharing is not applicable to this article.
